# Effect of pre-heating on the mechanical properties of silorane-based and methacrylate-based composites

**DOI:** 10.4317/jced.52807

**Published:** 2016-10-01

**Authors:** Narmin Mohammadi, Elmira Jafari-Navimipour, Soodabeh Kimyai, Amir-Ahmad Ajami, Mahmoud Bahari, Mohammad Ansarin, Mahsa Ansarin

**Affiliations:** 1 Associate professor at the Operative Dentistry Department, Dental and Periodontal Research Center, Faculty of Dentistry, Tabriz University of Medical Sciences, Tabriz, Iran; 2 Professor at the Operative Dentistry Department, Dental and Periodontal Research Center,Faculty of Dentistry, Tabriz University of Medical Sciences, Tabriz, Iran; 3 Assistant professor at the Operative Dentistry Department, Dental and Periodontal Research Center,Faculty of Dentistry, Tabriz University of Medical Sciences, Tabriz, Iran; 4 Student of the Biomedical Engineering Masters Program, Koç University, Istanbul, Turkey; 5 DDS, private practice, Tabriz, Iran

## Abstract

**Background:**

The use of composites in dental restoration has been commonly criticized, due to their underwhelming mechanical properties. This problem may be solved partially by preheating. The present research aims to determine the effect of preheating on the mechanical properties of two different classes of composites.

**Material and Methods:**

A Silorane-based (Silorane) and a Methacrylate-based (Z250) composite were preheated to different temperatures (25, 37, and 68 °C) and afterwards were tested with the appropriate devices for each testing protocol. The material’s flexural strength, elastic modulus, and Vickers microhardness were evaluated. Two-way ANOVA, and Tukey’s post hoc were used to analyze the data.

**Results:**

Microhardness and elastic modulus increased with preheating, while flexural strength values did not increase significantly with preheating. Furthermore the methacrylate-based composite (Z250) showed higher values compared to the Silorane-based composite (Silorane) in all the tested properties.

**Conclusions:**

Preheating Silorane enhances the composite’s microhardness and elastic modulus but does not affect its flexural strength. On the other hand, preheating Z250 increases its microhardness but does not change its flexural strength or elastic modulus. In addition, the Z250 composite shows higher microhardness and flexural strength than Silorane, but the elastic modulus values with preheating are similar. Therefore Z250 seems to have better mechanical properties making it the better choice in a clinical situation.

** Key words:**Composite, elastic modulus, flexural strength, microhardness, preheating.

## Introduction

In dental restorations, composite resins have underwhelming mechanical properties compared to amalgam; however, they do not contain mercury, a highly toxic substance found in amalgam compounds, and have higher aesthetic appeal. Consequently, their recently increased popularity as posterior filling materials remains controversial (1-3). Mechanical properties in resin composites are mainly related to the microstructure (type, size, number of filler particles) and the composition (2). Improved mechanical properties of resin composites along with good clinical performance have made them better suited for posterior restorations (4). Despite improved mechanical properties, clinical data shows that mass fracture is one of two main problems of composite restorations, the other being secondary decay (5).

Proper techniques should be implemented to improve mechanical properties and degree of conversion without sacrificing marginal adaptation of the composite. One recommended method is Soft-Start light polymerization (6), and the other that is getting more popular is preheating composites (7-10). A study found that by preheating composite to 60 degrees Celsius before curing, the degree of conversion from surface to depth increases by 2 mm (9).

Other studies revealed preheating resin composites before placing in the cavity and shaping, increases adaptation with the prepared walls by lowering viscosity and increasing flow, thus reducing micro leakage and increasing restoration durability (11-14).

Preheating composite before placing in the cavity increases radicals and monomers (9,15), and improves polymerization (5) thereby increasing the crosslinking of the polymer network and improving mechanical and physical properties (10). Furthermore preheating increases the surface hardness and the cure depth of composites (16,17). The polymerization rate of resin composites is 50-75%, which directly affects mechanical properties, and therefore restoration longevity (18).

Deb *et al.* report higher flexural strength after preheating of the studied composites (18). However, two other studies show no significant difference in flexural strength between preheated and non-heated composites (19,20). Other studies on surface hardness of composites after heating show heating before cure increases surface hardness of some composites (17,20,21). On the other hand, heating other composites before curing does not increase the surface hardness (20). Aforementioned studies show that depending on composite type and different compositions of composites, heating them before curing results in different outcomes in the mechanical properties of composites.

Silorane based composites are a new type of composites introduced by 3M-ESPE. These composites are made from silorane monomers. Silorane is made of a combination of Siloxane and Oxirane. The polymerization mechanism of silorane is cationic ring opening and is different from methacrylate based composites which are polymerized by free radical based polymerization (22,23). Considering the different composition and polymerization mechanisms of these composites from methacrylate composites, the question that comes to mind is whether preheating could affect the mechanical properties of these composites? Would it improve the mechanical properties of these composites or will it show negative effects on Silorane-based composites? The aim of the current study is to study the effect of preheating on mechanical properties (flexural strength, microhardness, and modulus of elasticity) of two different classes of composites.

## Material and Methods

Our goal in this study was to examine how preheating affects the mechanical properties of two composites, one silorane-based and one methacrylate-based. We evaluated three mechanical properties (flexural strength, modulus of elasticity, and microhardness,) of the two composites preheated to 3 different temperatures (25, 37, and 68 °C). The two composites used were Filtek Silorane in the shade A2 (3M-ESPE, Dental product, St. Paul, MN, USA) and Filtek Z250 in the shade A2 (3M-ESPE, Dental product, St. Paul, MN, USA). The resulting data was evaluated statistically by Two-Way ANOVA followed by Tukey’s Post Hoc for pairwise comparisons.

The study protocol was approved by the local research ethics committee.

-Flexural Strength and Elastic Modulus

We first evaluated the two composites’ flexural strength. To prepare samples for this test, we first placed each composite in a thermostatically controlled water bath for 15 minutes which had been set to the specific preheating temperature (25 or 37 or 68 °C). Afterwards, we packed the composite into a 25mm×2mm×2mm mold and placed a Mylar strip on top and then a glass slide on top of that to remove excess material. We then cured the composite with a visible light curing device called Demetron A.2 (Kerr Dental Equipment) at an intensity of 1000 mW/cm2 for 40 seconds. Each composite (Z250 or Silorane) was warmed to either 25, 37, or 68 °C resulting in 6 groups. We made 17 samples for each group, with a total of 102 samples.

After preparing the samples, a three-point bending test was done with the Universal Testing Machine (Hounsfield Test Equipment, Model H5KS-Surray-UK) at a crosshead speed of 0.5mm/min and the flexural strength was found using the formula (20): σ = 3FL/2bh2.

Where F is the load (force) at the fracture point (N), L is the length of the support span (mm), b is width of the sample (mm), and h is thickness of the sample (mm).

To evaluate the material’s elastic modulus, we used the maximum load and deflection attained from the flexural strength testing. These numbers were placed into the following formula to give us the elastic modulus in each sample (20): E = FL3/4bh3d.

Where d is the deflection (in millimeters) corresponding to the maximum load F. The data obtained from the experiment was then evaluated by Two-way ANOVA followed by Tukey’s Post Hoc analysis where it was applicable.

-Vickers Microhardness

We then evaluated the microhardness of the composites. The composites tested were Filtek Silorane in the shade A2 (3M-ESPE, Dental product, St.Paul, MN, USA) and Filtek Z250 in the shade A2 (3M-ESPE, Dental product, St.Paul, MN, USA). We placed the composites in a similar water bath set to the specific preheating temperature (25 or 37 or 68 degrees Celsius) for 15 minutes. Afterwards, the composite was placed into a mold with a diameter of 4mm and a thickness of 2mm; a Mylar strip and a glass slide were placed on top to remove excess material. It was then cured with a visible light curing device named Demetron A. 2 (Kerr Dental Equipment) at an intensity of 1000 mW/cm2 for 40 seconds. Similar to the flexural strength experiment, we made a total of 102 samples, with 17 for each composite at each temperature.

To determine the microhardness of the prepared samples, we used a Microhardness tester (UH, VMHS, AUTO, WALTER UHL, Technische Mikroskopie GMbH Co.KG-Loherstrabe, Germany) with a Vickers indenter with a 300gr load for 10 seconds (17). The resulting data was then evaluated statistically by Two-Way ANOVA followed by Tukey’s Post Hoc for pairwise comparisons.

## Results

**Figure 1:**
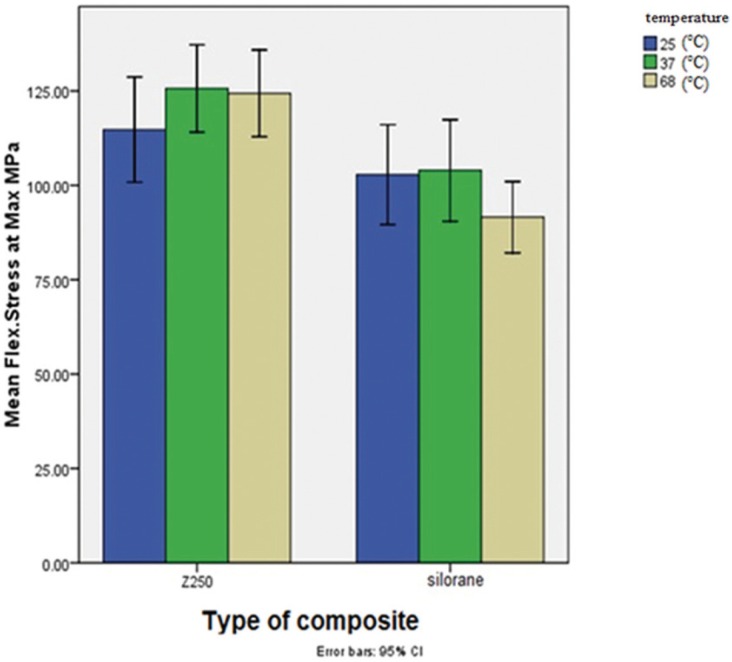
Mean flexural strength of Z250 and Silorane composites at different preheating temperatures (Error Bars = 1 Standard Deviation).

**Figure 2:**
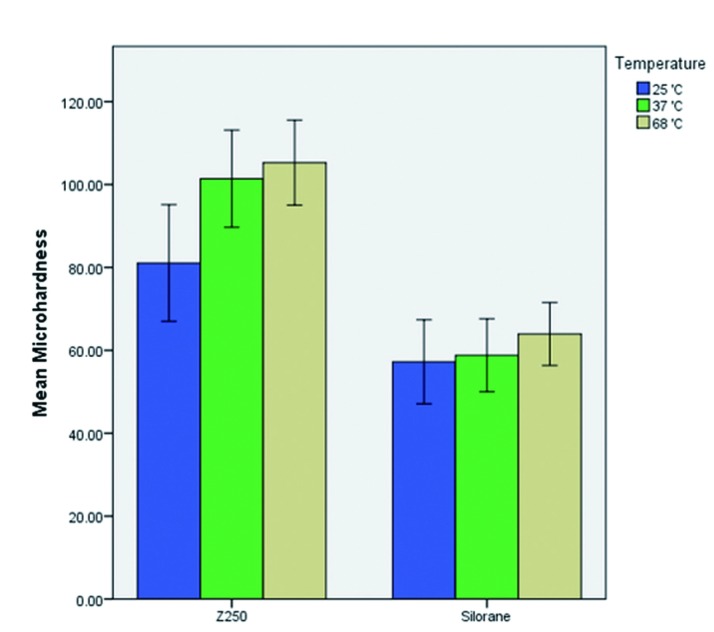
Mean microhardness of Z250 and Silorane composites at different preheating temperatures (Error Bars = 1 Standard Deviation).

We conducted statistical analysis by Two-Way ANOVA on the results of the mechanical experiments. The analysis showed that the Z250 composite had higher flexural strength than Silorane (*p*<0.005) ( Table 1, Fig. 1). However, Mean flexural strength did not have a statistically meaningful dependency on the preheating temperature (F2, 96=0.83, *p*=0.43). The Z250 composite also had higher microhardness values (*p*<0.0005) ( Table 2, Fig. 2).

**Table 1:**
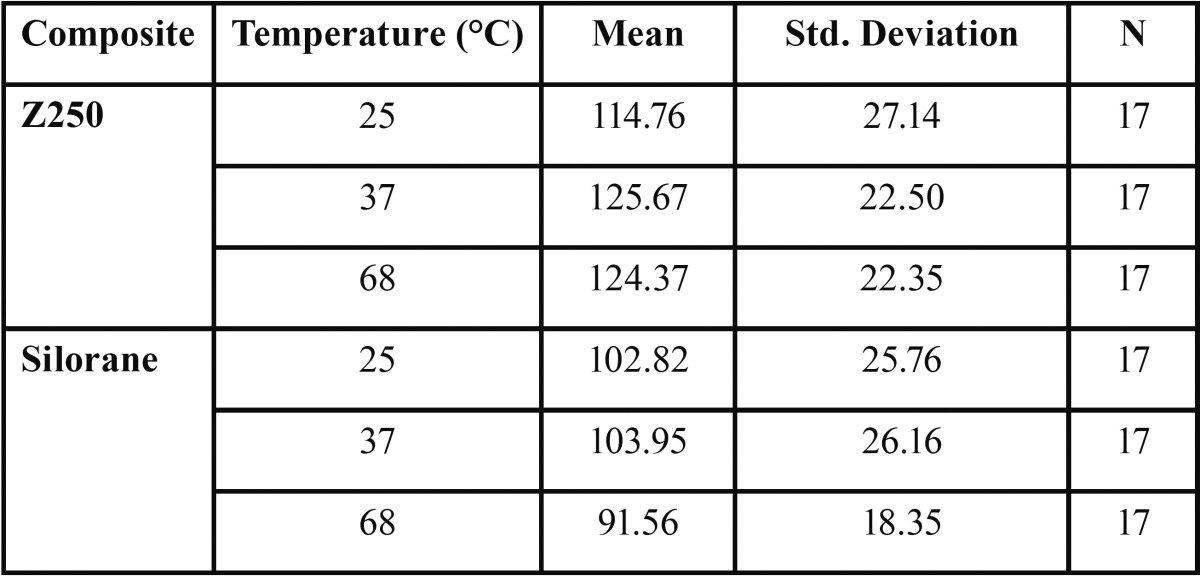
Mean and Standard Deviation of flexural strength (MPa) in the groups and subgroups of the study.

**Table 2:**
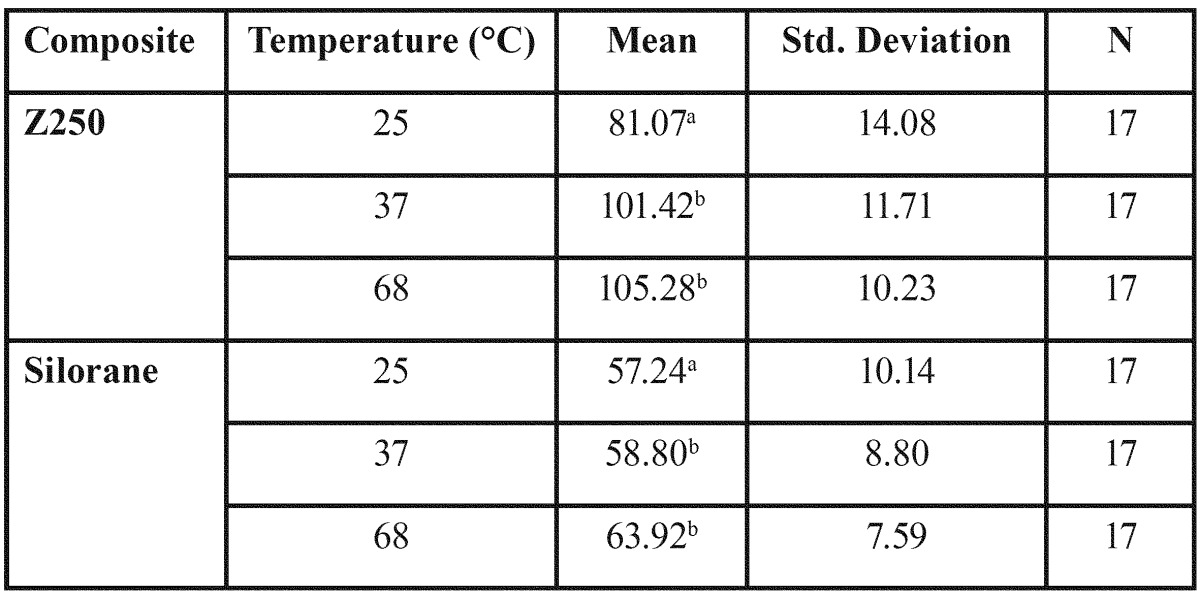
Mean and Standard Deviation of Microhardness (Vickers’s Hardness) in the groups and subgroups of the study.

**Figure 3:**
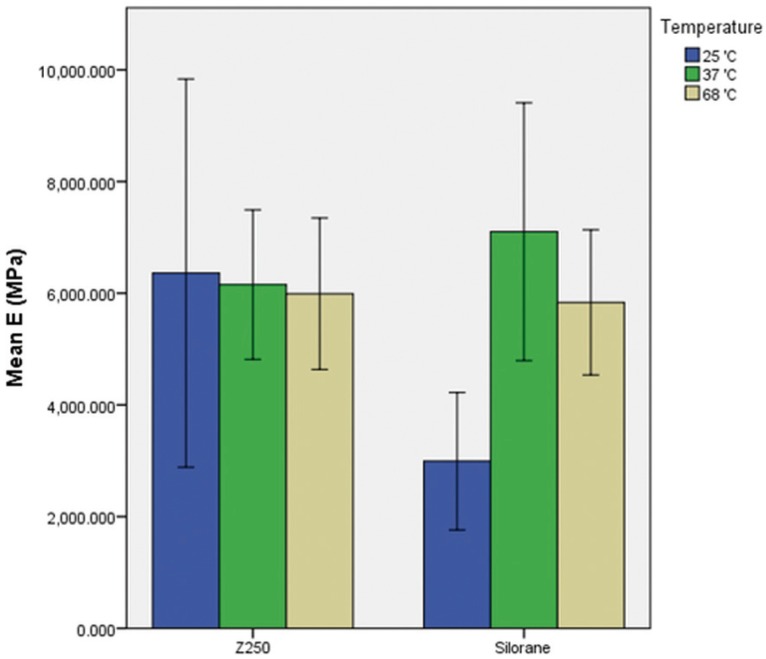
Mean Elastic Modulus of Z250 and Silorane composites at different preheating temperatures (Error Bars = 1 Standard Deviation).

Regarding the elastic modulus, the Z250 composite’s modulus was found to be higher than that of Silorane (*p*=0.015) ( Table 3, Fig. 3). We also observed a statistically significant difference in elastic modulus values between preheating temperatures (F2, 96=8.21, *p*=0.001). Our cross analysis between the type of composite and the different temperatures showed significant statistical difference (F2, 96=10.57, *p*<0.001). Silorane’s elastic modulus significantly increased with preheating. However, Z250’s elastic modulus did not show any change.

**Table 3:**
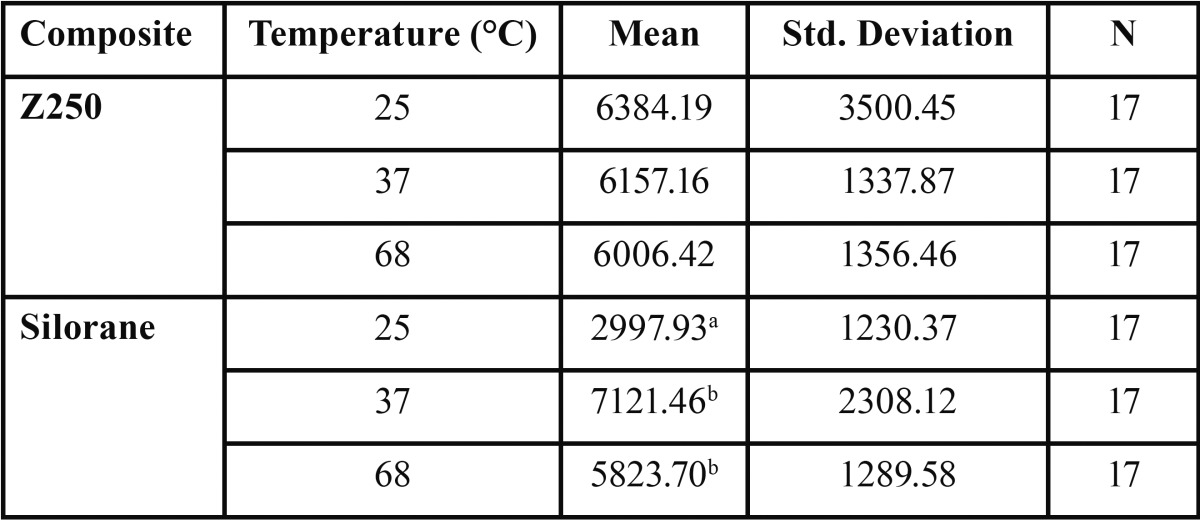
Mean and standard deviation of elastic modulus (MPa) in the groups and subgroups of the study.

A Tukey analysis test showed the 37 °C group had higher elastic modulus values than the 25°C group (*p*<0.0005). Also between the 25 °C and 68 °C groups there was a statistically significant difference (*p*=0.038) where the elastic modulus values for 68 °C were higher. We saw no statistical difference between the 37 °C and 68 °C groups (*p*=0.43).

Microhardness also significantly varied across preheating temperatures (F2, 96=18.98, *p*<0.001). Based on the results of a Tukey analysis test, the 37 °C groups had higher microhardness values than the 25°C groups (*p*<0.001). Also, microhardness values of the 68 °C group were higher than those of the 25°C groups (*p*<0.001). Between the 37 °C and 68 °C groups there was no significant statistical difference (*p*=0.25).

## Discussion

In this study, we evaluated how preheating affects the mechanical strength of two different composites (Z250 and Silorane). To do this, we conducted flexural strength and microhardness tests on 2 different composites, each preheated to 25, 37, or 68°C. We also calculated elastic modulus values from the flexural strength test and used all 3 mechanical property values to compare how the preheating treatment had affected the samples. In the following paragraphs, we will consider each mechanical property separately and compare our results with previous findings.

We found that preheating does not significantly affect the flexural strength of Z250 or Silorane. Previously, Uctasli *et al.* preheated Z250 and Grandio to different temperatures (25, 40, 45, 50 degrees Celsius) and tested their flexural strength and modulus. They found Z250 had higher values of flexural strength and modulus at all the tested temperatures compared to Grandio; in addition, they observed no significant correlation between flexural strength and preheating of the two composites and also between flexural modulus and preheating. Uctasli *et al.* concluded that the mechanical properties of resin composites are related to their filler content, where the composite with the highest volume of filler has the highest flexural strength and flexural modulus (20). In Deb *et al.’s* study the only composites that showed a significant increase in flexural strength after preheating were Spectrum TPH and F2000. Their other composites, Herculite, Heliomolar, Filtek P60, and Wave, didn’t show significant differences in flexural strength after preheating compared with their ambient state. They believe that the increase in the flexural strength for Spectrum TPH and F2000 may have been due to the increase in molecular mobility in the polymer system and hence better cross-linking in the polymer chains (18). There was a strong negative correlation between flexural strength and shrinkage for ambient and preheated conditions in Deb *et al.’s* study (18), so we can expect a composite with low shrinkage like Silorane to have higher flexural strength. However, another factor affecting strength is filler load. Deb *et al.* reported that the purpose of increasing the inorganic filler in a composite is to reinforce the organic matrix (18). Z250 has a higher filler percentage (82%) compared with Silorane (76%) and this fact may be responsible for the superior flexural strength of Z250. Since pre-heating appears to not affect flexural strength we believe that the filler load is the only factor that can affect the flexural strength of composites.

In a comparison of the two composite’s elastic moduli, we found Z250’s elastic modulus to be significantly higher than Silorane’s. This may be a consequence of the higher filler loading percentage in Z250 compared to Silorane. It may also be due to the different polymerization mechanisms where Silorane is polymerized by cationic ring opening and Z250 is polymerized by free radical addition polymerization of the corresponding methacrylate monomers. We found that preheating Silorane significantly affects its elastic modulus, but preheating Z250 showed no such effect.

In this study, the microhardness of both Z250 and Silorane increased with preheating. Nada *et al.* (21) found similar results with Z100 and Clearfil Majesty. They speculate their surface hardness increase may have been due to a higher rate of conversion (caused by higher temperature) that resulted in highly cross-linked networks. However, the increase in microhardness was only consistent in Z100 and Clearfil Majesty and not for Light Core specimens. Nada *et al.* (21) wrote that this is most likely due to the different chemical composition of these composites, specifically the monomer nature. Z100 and Clearfil Majesty both contain TEGDMA in their Bis-GMA based monomers, but Light Core does not. Increasing the amount of TEGDMA in the monomer increases the resin composite’s degree of conversion. Nada *et al.* (21) also report that filler mass fraction influences a composite’s surface and mechanical properties. Filler loading must be high in order to support the highly cross-linked networks formed after preheating a composite (21). This could be another reason for the higher microhardness of Z250 (82% filler loading) compared to Silorane (76% filler loading). Another factor that could affect the microhardness of the composites is the composite cooling before being placed in the molds. A study reported a 50% drop in temperature within 2 minutes of removing composite from the composite campule. Positive outcomes in testing also depend on the brand of composite and the type (21). Munoz *et al.* reported that preheating hybrid and microhybrid composites increased the hardness and depth of curing (using both Halogen and LED curing lights) (16). In another study it was found that an increased temperature decreased the composites viscosity and enhanced radical mobility resulting in higher conversion rates and a harder composite (7). Similar to our results with the 37°C and 68°C preheated samples, Munoz *et al.* found that there was little difference in surface hardness between polymerizing the composite at 60°C compared to 37.7°C (16). Daronch *et al.* reported a correlation between surface microhardness and degree of polymerization, suggesting that increases in VHN values may have been due to better polymerization at higher temperatures (9,10). Since after preheating the composite is to be placed in contact with the tooth, pulpal damage is a primary concern. A study found that when placing a composite preheated to 60°C into a prepared tooth, the temperature increased 7.9°C (16). We must note that elastic modulus is known to represent the elastic mechanical response and flexural strength represents a material’s resistance to cracking. But microhardness measures, such as the Vickers indentation test, are known to gauge a material’s resistance to plastic deformation. Neither composite displays an increase in flexural strength with increases in preheating temperature, so we can assume the strength against cracking does not improve. On the other hand, both composites show an increase in Vickers microhardness with an increase in preheating temperature; we believe that this shows the composite’s resistance to plastic deformation is increasing. This has positive implications for posterior tooth restorations.

In this study, we evaluated a few mechanical properties of composites after pre-heating. Further research is needed to evaluate the other mechanical properties and whether or not they are influenced by preheating. We conducted this study in vitro without the use of any dental tissue. Experimental results may differ in a clinical situation. It may be helpful to do testing on the composites and dental tissue to determine the effect of preheating on the dental tissue and on the pulp.

In conclusion, preheating Silorane enhances the composite’s microhardness and elastic modulus but does not affect its flexural strength. On the other hand, preheating Z250 increases its microhardness but does not change its flexural strength or elastic modulus. In addition, the Z250 composite shows higher microhardness and flexural strength than Silorane, but the elastic modulus values with preheating are similar.

## References

[1] Christensen GJ (2010). Should resin-based composite dominate restorative dentistry today?. J Am Dent Assoc.

[2] Al-Sharaa KA, Watts DC (2003). Stickiness prior to setting of some light cured resin-composites. Dent Mater.

[3] Manhart J, Kunzelmann K H, Chen H, Hickel R (2000). Mechanical properties and wear behavior of light-cured packable composite resins. Dent Mater.

[4] Cramer NB, Stansbury JW, Bowman CN (2011). Recent advances and developments in composite dental restorative materials. J Dent Res.

[5] Sarrett DC (2005). Clinical challenges and the relevance of materials testing for posterior composite restorations. Dent Mater.

[6] Watts D, Hindi AA (1999). Intrinsic 'soft-start' polymerisation shrinkage-kinetics in an acrylate-based resin-composite. Dent Mater.

[7] Lovell LG, Lu H, Elliott JE, Stansbury JW, Bowman CN (2001). The effect of cure rate on the mechanical properties of dental resins. Dent Mater.

[8] Lovell LG, Newman SM, Bowman CN (1999). The effects of light intensity, temperature, and comonomer composition on the polymerization behavior of dimethacrylate dental resins. J Dent Res.

[9] Daronch M, Rueggeberg F, De Goes M (2005). Monomer conversion of pre-heated composite. J Dent Res.

[10] Daronch M, Rueggeberg F, De Goes M, Giudici R (2006). Polymerization kinetics of pre-heated composite. J Dent Res.

[11] Choudhary N, Kamat S, Mangala TM, Thomas M (2011). Effect of pre-heating composite resin on gap formation at three different temperatures. J Conserv Dent.

[12] Ahn KH, Lim S, Kum KY, Chang SW (2015). Effect of preheating on the viscoelastic properties of dental composite under different deformation conditions. Dent Mater J.

[13] Wagner W, Aksu M, Neme A, Linger J, Pink F, Walker S (2008). Effect of pre-heating resin composite on restoration microleakage. Oper Dent.

[14] Elsayad I (2009). Cuspal movement and gap formation in premolars restored with preheated resin composite. Oper Dent.

[15] El-Korashy D (2010). Post-gel shrinkage strain and degree of conversion of preheated resin composite cured using different regimens. Oper Dent.

[16] Munoz CA, Bond PR, Sy-Munoz J, Tan D, Peterson J (2008). Effect of pre-heating on depth of cure and surface hardness of light-polymerized resin composites. Am J Dent.

[17] Lucey S, Lynch C, Ray N, Burke F, Hannigan A (2010). Effect of pre-heating on the viscosity and microhardness of a resin composite. J Oral Rehabil.

[18] Deb S, Di Silvio L, Mackler HE, Millar BJ (2011). Pre-warming of dental composites. Dent Mater.

[19] Fróes-Salgado NR, Silva LM, Kawano Y, Francci C, Reis A, Loguercio AD (2010). Composite pre-heating: effects on marginal adaptation, degree of conversion and mechanical properties. Dent Mater.

[20] Uctasli MB, Arisu HD, Lasilla LV, Valittu PK (2008). Effect of preheating on the mechanical properties of resin composites. Eur J Dent.

[21] Nada K, El-Mowafy O (2011). Effect of precuring warming on mechanical properties of restorative composites. Int J Dent.

[22] Amirouche-Korichi A, Mouzali M, Watts DC (2009). Effects of monomer ratios and highly radiopaque fillers on degree of conversion and shrinkage-strain of dental resin composites. Dent Mater.

[23] Gonçalves F, Azevedo CL, Ferracane JL, Braga RR (2011). BisGMA/TEGDMA ratio and filler content effects on shrinkage stress. Dent Mater.

